# Systematic or targeted molecular screening of SHOX gene variations in children with idiopathic short stature?

**DOI:** 10.1210/jendso/bvag054

**Published:** 2026-03-17

**Authors:** Romee Pouliquen, Sebastien Schmitt, Tifenn Gueguen, Aurelie Donzeau, Stephanie Rouleau, Fabienne Emeriau, Natacha Bouhours-Nouet, Lucie Levaillant, Regis Coutant

**Affiliations:** Department of Pediatric Endocrinology, University Hospital, 49100 Angers, France; Reference Center for Rare Pituitary Diseases, University Hospital, 49100 Angers, France; Department of Medical Genetics, University Hospital, 49093 Nantes, France; Department of Pediatric Endocrinology, University Hospital, 49100 Angers, France; Reference Center for Rare Pituitary Diseases, University Hospital, 49100 Angers, France; Department of Pediatric Endocrinology, University Hospital, 49100 Angers, France; Reference Center for Rare Pituitary Diseases, University Hospital, 49100 Angers, France; Department of Pediatric Endocrinology, University Hospital, 49100 Angers, France; Reference Center for Rare Pituitary Diseases, University Hospital, 49100 Angers, France; Department of Pediatric Endocrinology, University Hospital, 49100 Angers, France; Reference Center for Rare Pituitary Diseases, University Hospital, 49100 Angers, France; Department of Pediatric Endocrinology, University Hospital, 49100 Angers, France; Reference Center for Rare Pituitary Diseases, University Hospital, 49100 Angers, France; Department of Pediatric Endocrinology, University Hospital, 49100 Angers, France; Reference Center for Rare Pituitary Diseases, University Hospital, 49100 Angers, France; Department of Pediatric Endocrinology, University Hospital, 49100 Angers, France; Reference Center for Rare Pituitary Diseases, University Hospital, 49100 Angers, France

**Keywords:** idiopathic short stature, dyschondrosteosis, Leri–Weill syndrome, SHOX, screening, children

## Abstract

**Context:**

Diagnosing SHOX gene variations is important because growth hormone treatment is an approved option for affected children. Subtle clinical and radiological abnormalities were reported in SHOX deficiency associated with idiopathic short stature (ISS). Whether systematic or phenotype-based molecular screening should be performed remains debated.

**Objective:**

To determine whether simple radiological features on left-hand radiography could serve as indicators for molecular analysis of the SHOX gene, and to compare these with published clinical/radiological scores.

**Methods:**

This retrospective study included 266 patients diagnosed with ISS who underwent SHOX gene analysis without any predefined selection criteria at the Pediatric Endocrinology Unit of Angers University Hospital from 2016 to 2023, aiming to determine the rate of SHOX gene variations. We also included 33 ISS patients diagnosed with a SHOX gene variation between 2005 and 2015 to refine sensitivity analyses.

**Results:**

Systematic screening using MLPA identified SHOX gene variations in 9.8% of ISS children and sequencing in MLPA-negative subjects detected an additional 6%. Variations occurred in the coding regions in one-third and in the enhancer regions in two-thirds. A cutoff of 147° for the convexity of the distal radial metaphysis showed sensitivity/specificity of 89%/50%. A cutoff of 128° for pyramidalization of the carpal row yielded sensitivity/specificity of 86%/49%. Combining both criteria yielded 91% sensitivity and 70% specificity. Previous scores proposed by Rappold and Binder had sensitivity/specificity of 36%/51% and 81%/10%, respectively.

**Conclusion:**

Systematic molecular screening by MLPA and sequencing is recommended to detect all SHOX gene variants in children with ISS.

A proportion of short stature is caused by monogenic disorders that lead to skeletal dysplasia. Among the molecular causes of skeletal dysplasia, heterozygous variations in the SHOX gene were first identified in 1997 as the primary cause of Leri–Weill syndrome [1, 2]. It is characterized by disproportionate short stature with mesomelic shortening of the limbs and is associated with spontaneous subluxation of the distal ulna forward (Madelung deformity). Other dysmorphic signs have been described, including micrognathia, high-arched palate, scoliosis, shortening of the fourth and fifth metacarpals, and muscular hypertrophy [3-6]. Radiological features typically linked to SHOX gene variation include radial bowing, enlarged diaphysis of the radius, triangularization of the radial epiphysis, pyramidalization of the carpal row, dislocation of the ulna at the wrist and elbow, shortened fourth and fifth metacarpals, and lucency along the ulnar border of the distal radius [7-9]. The benefits of rhGH for increasing height in patients with SHOX gene variations have been proven, especially when initiated early, and the FDA and EMEA have granted approvals [8, 10-13]. This highlights the importance of diagnosing SHOX gene variation. Following the initial description of SHOX gene variations in dyschondrosteosis, they were primarily investigated in short individuals exhibiting distinctive clinical disproportion and radiological findings, which resulted in a high molecular yield.

To further refine the indications for SHOX molecular studies, Rappold published a scoring system in 2007 based on eight clinical criteria, while Binder published an algorithm in 2011 that focused on clinical features and 3 radiological characteristics derived from left hand and wrist radiography, the triangularization of the distal radial epiphysis, the pyramidalization of the carpal row, and the distal radius lucency [5, 6]. In addition to these radiological criteria, Vannelli et al [8] proposed to include the convexity of the distal radial metaphysis, but no cutoffs were provided for comparisons [9]. The search for SHOX gene variations in subjects with apparently isolated short stature (ISS), defined as short stature with no other specific findings or body disproportion, revealed that they were present in 1.7% to 22% of the subjects [4-7, 9, 14-18]. Several of these studies do not clarify whether affected subjects had obvious, subtle, or no clinical and radiological abnormalities, and this may contribute to the wide variability in the frequency of SHOX gene variation among children with ISS [4-7, 9, 14-18]. Additionally, the variability in the frequency may also result from the different molecular tools used across studies, which detected various types of variations in the SHOX gene. The studies by Binder identified large SHOX gene deletions only in coding regions [5, 18]; the study by Rappold identified large and small deletions as well as point mutations only in coding regions [6]; other studies identified specific deletions and point mutations within the coding regions [14]; and some also targeted enhancer and coding regions, finding deletions, duplications, and point mutations [7, 9, 15-17].

Given the diversity of phenotypes, it is unclear whether the criteria for molecular screening of SHOX gene variation in short children should still depend on specific clinical and/or radiological findings. In light of the reported prevalence of SHOX gene variation in ISS and the associated phenotypic variability, we expanded our molecular investigation of SHOX gene variation from 2016 to include all children referred to our center for short stature that remained unexplained after a diagnostic workup (266 of 568 referred children). Since the SHOX protein is involved in growth plate physiology [7], it is conceivable that subtle radiological indices may still guide toward SHOX molecular screening: the goal of this study was to determine whether simple radiological features (distal radial metaphysis convexity angle, carpal row pyramidalization angle, triangularization index of the distal radial epiphysis, and distal radial lucency) observed in left-hand and wrist radiography (for bone age assessment) were linked to the presence of SHOX gene variations and could serve as reliable indicators for molecular analysis of the SHOX gene. To achieve this objective, we compared the radiological features of affected and unaffected individuals. To enhance the study's discriminating power, we also included subjects with SHOX gene variations diagnosed before our systematic screening policy, between 2005 and 2015. We also examined the discriminating ability of these indices based on whether SHOX gene variations occurred in the coding and enhancer regions. Finally, we compared these indices to the clinical scoring system from Rappold et al and the clinical and radiological decision algorithm from Binder et al [5, 6].

## Methods

### Patients

**Figure 1:**
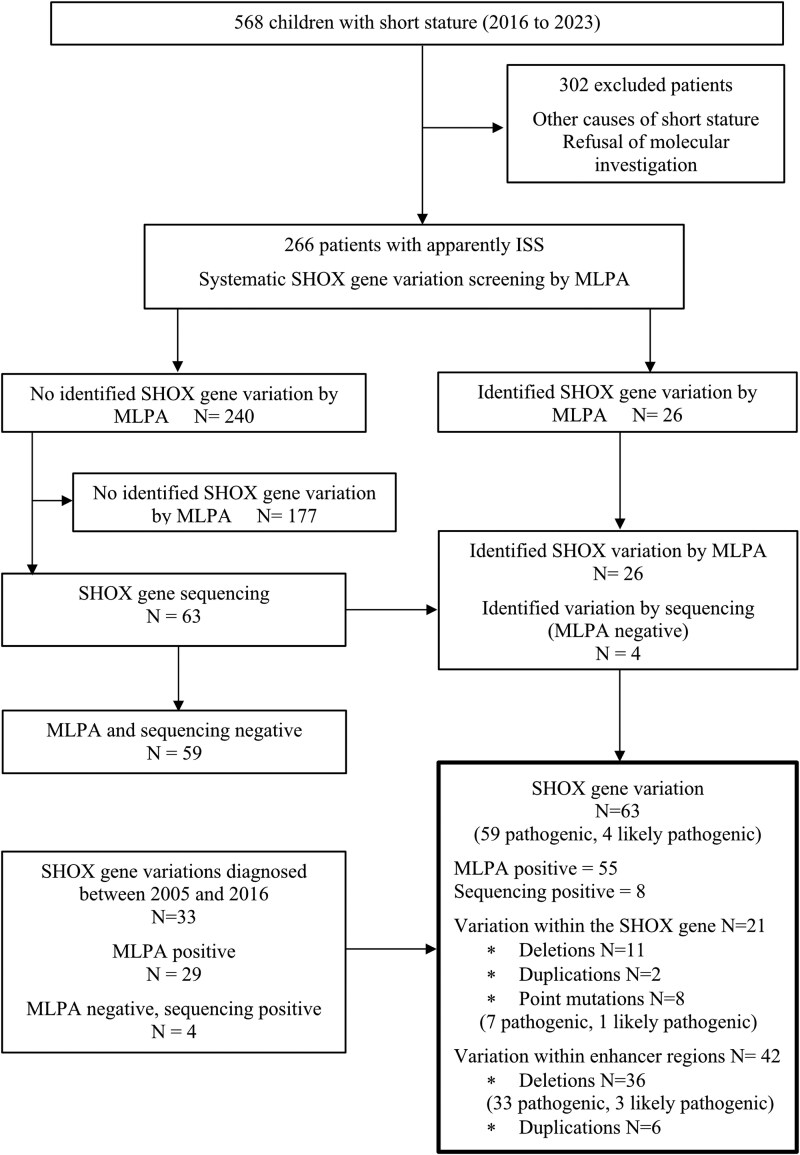
Flow chart of the study.

This study is a descriptive, retrospective, and noninterventional analysis based on clinical and radiological data from patients referred to the Pediatric Endocrinology Unit of Angers University Hospital for short stature between 2016 and 2023. The exclusion criteria included syndromic, endocrine, and nutritional causes of short stature, skeletal dysplasia evident from the morphotype and radiological analyses, chronic diseases or treatments affecting growth, and the absence of SHOX gene analysis. From 2016 to 2023, 568 children were referred to our center for short stature, of which 302 were excluded because the diagnostic workup identified other causes for short stature, or the families refused the molecular investigation. A total of 266 children with a final diagnosis of ISS underwent SHOX gene molecular analysis, with 30 identified as carrying a SHOX gene variation. To further assess the sensitivity of radiological criteria for the molecular diagnosis of SHOX gene variations, we also included patients diagnosed with a SHOX gene variation between 2005 and 2015; 33 additional patients were added (see Flowchart, Fig. 1). Before 2016, the criteria for SHOX gene screening in ISS included unexplained very short stature (<−3 SD score) and/or familial short stature (paternal and/or maternal height < −2 SD score); subjects with evident skeletal dysplasia, including Leri–Weill phenotype, were excluded from the present study. The parents or legal guardians provided written consent for the molecular study of the SHOX gene. Collecting personal data from medical records for research requires only the nonopposition of children and their families, in accordance with French regulation (“loi Jardé”). The ethics committee of Angers University Hospital approved the study (number 2024-043). The anonymous database was reported to the French Data Protection Authority (CNIL) under number ar24-0003v0.

Demographic and morphometric characteristics were collected for each patient, including gender, chronological age, neonatal length, height, body mass index, sitting height to height ratio, arm span to height ratio, parents' heights, the presence of short stature in the family (defined as height below 152 cm for women and below 163 cm for men), and radiological characteristics of the left hand and wrist (see below). The arm span to height and sitting height to height ratio cutoff values proposed by Rappold were 0965 and 0.555, respectively [6]. For each patient, we calculated the score described by Rappold based on the following criteria: arm span/height ratio, sitting height/height ratio, body mass index, cubitus valgus, short forearm, bowing of the forearm, muscular hypertrophy, and dislocation of the ulna at the elbow. This score, consisting of 8 items, was based on data from 1608 short individuals: 68 subjects had a SHOX gene variation in the coding region (16 point mutations and 42 deletions); no search for variation in the enhancer regions was conducted, which could have led to misclassification of some SHOX-negative subjects [5]. We also determined whether our patients would have been tested for the SHOX gene variation, considering the algorithm described by Binder: exclusion of alternative cause of short stature, presence of Madelung deformity or first-degree relative with a SHOX deficiency, extremities-trunk ratio < −1SDS, presence of muscular hypertrophy or minor abnormalities such as shortening of the fourth of fifth metacarpals, high arched palate, increased carrying angle of the elbow, scoliosis and micrognathia, presence of typical hand X-ray abnormalities defined as exaggerated triangularization of the radial epiphysis, exaggerated pyramidalization of the carpal row, and exaggerated lucency of the distal radius. This algorithm did not consider measurements but rather the general aspects of radiography (presence or absence of the radiological features) and was based on data from 145 short individuals: 8 children had a SHOX gene deletion in the coding region; no search for point mutation within the coding region or variation in the enhancer regions was conducted, which could have led to misclassification of some SHOX-negative subjects [5, 18].

### Radiological characteristics of the left hand and wrist

**Figure 2:**
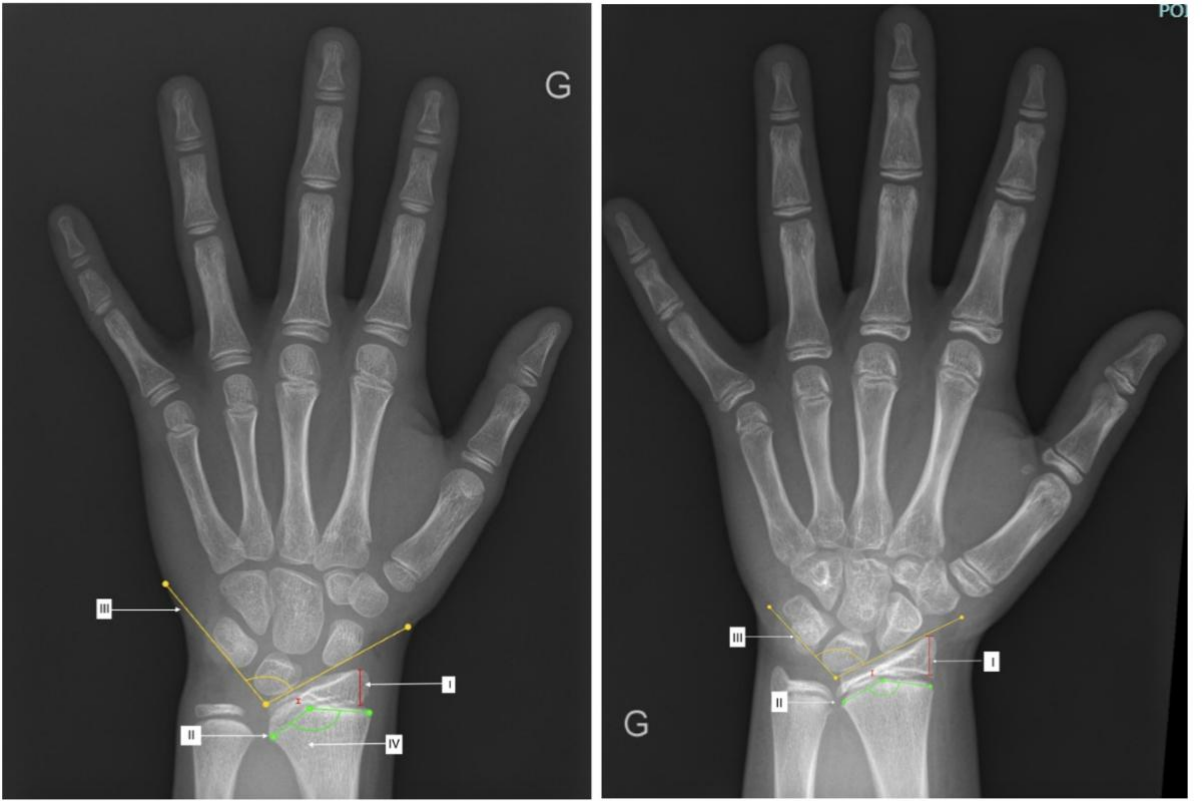
Radiological criteria in SHOX variation. I. Triangularization of the distal radial epiphysis is defined as the ratio of the maximum lateral width to the minimum width. II. The convexity angle of the distal radial metaphysis is evaluated by locating the 2 lateral points and the top point on the distal radial metaphysis, and then drawing lines between the top point and each lateral point. III. The pyramidalization angle of the carpal row is defined by the intersection of 2 lines: (i) the tangent line to the lunate and the triquetrum and (ii) the tangent line to the lunate and the scaphoid. IV. Increased lucency of the ulnar border of the distal radius.

Four characteristics were analyzed: the triangularization index of the distal radial epiphysis (defined as the width ratio of the maximal lateral width to the minimal width), the convexity of the distal radial metaphysis (an angle defined by the 2 lateral points and the top point of the metaphysis), the pyramidalization of the carpal row (the carpal angle defined by the intersection of the tangent to the lunate and the triquetrum with the tangent to the lunate and the scaphoid), and the lucency of the ulnar border of the distal radius (Figure 2). All measurements were conducted using the software Image J (the National Institutes of Health, Bethesda, MD, USA). Intra- and inter-observer reproducibility was evaluated for 30 anonymized X-rays, assessed by the same observer twice (intra-observer reproducibility) and by 2 different observers (inter-observer reproducibility). Both intra- and inter-observer agreements were satisfactory, with Cronbach's alpha values of 0.921 and 0.942, respectively.

### Genetic analysis

Molecular analysis of the gene and its enhancer regions was performed at the Genetic Laboratory of Nantes University Hospital using 2 different methods: MLPA (Multiplex Ligation-dependent Probe Amplification) and sequencing. MLPA analysis was conducted with the SALSA MLPA Probemix kit P018 G1 and G2 (MRC-Holland), analyzing the 6 exons and enhancer regions located 5′ and 3′. Sequencing was performed using the Sanger method on ABI Applied Biosystems 3130XL sequencers, analyzing all coding exons, intron-exon junctions, and enhancer elements found either 5′ or 3′. Most instances of SHOX haploinsufficiency were reported to result from deletions (around 80%), and 18% from point mutations [3]. Therefore, MLPA, which can detect microdeletions and duplications, is the technique with the highest sensitivity [19]. It is relatively low cost and requires only a small amount of DNA samples [20]. We did not perform sequencing in all cases of negative MLPA analyses, as it is time-consuming and costly, but in a subset of subjects (see flow chart, Fig. 1, and statistical analysis below). The list of identified SHOX gene variants and their pathogenicity is provided in Tables 1 and 2. Pathogenicity was confirmed if the variation involves the entire SHOX gene, has been previously linked to the Leri-Weill phenotype, or causes skeletal effects in experimental models (reviewed by Marchini et al) [7].

**Table 1:** Identified SHOX gene variants and their pathogenicity (coding region)

Heterozygous SHOX gene variation involving the coding regions	*N*	Pathogenicity
Deletion of 26 contiguous probes (013141_L20651 to 13597_L15055) in the PAR1 region carrying the entire SHOX gene and its 3′ regulatory elements (heterozygous)	1	Pathogenic
Deletion of 19 contiguous probes (01341_L20651 to 06293-L20177) in the PAR1 region carrying the entire SHOX gene and the 3′ regulatory region downstream of the SHOX gene (CNE2, CNE3, CNE4, CNE5, CNE7, CNE9)	5	Pathogenic
Deletion of 12 contiguous probes (18891_LL25088 to 13821_L14642) in the PAR1 region, carrying the entire SHOX gene, the 5′ regulatory region, and the 3′ regulatory region downstream of the SHOX gene (CNE2, CNE3, CNE4, CNE5, CNE7, CNE9)	1	Pathogenic
Deletion of 4 contiguous probes (01146_L06220 to 01149_L19676) corresponding to exons 2 to 5 of the gene	3	Pathogenic
Deletion of 3 contiguous probes including exon 6 of the SHOX gene	1	Pathogenic
Presence of 2 distinct rearrangements in the PAR1 region: a copy number greater than 3 for the first 2 contiguous probes in the region studied (probes 09333_L10292 and 18889_L25087) including the regulatory element CNE5 located in 5′, and more than 3 copies for a region bounded by 4 contiguous probes (01148_L15501 to 09336_L20178) from exon 4 to intron 6 of the gene	1	Pathogenic
Duplication of 16 contiguous probes in the PAR1 region including the entire SHOX gene (probe 18889-L25087 to 13296-L20175)	1	Pathogenic
c.655G>C (p.Glu219Gln) variant in exon 4 of the SHOX gene in a heterozygous state	1	Likely pathogenic
c.394T>G in exon 3 leading to the missense variant p.Leu132Val.	2	Pathogenic
c.464G>T in exon 4 leading to the missense variant p.Gly155Val	2	Pathogenic
c.518G>A in exon 4 leading to the missense variant p.Arg173His	1	Pathogenic
c.634_635del in exon 6 leading to the frameshift mutation (p.Val212Profs*178)	2	Pathogenic

Pathogenicity was confirmed if the variation involves the entire SHOX gene, has been previously linked to the Leri–Weill phenotype, or causes skeletal effects in experimental models (reviewed by Marchini et al) [7, 21].

**Table 2:** Identified SHOX gene variants and their pathogenicity (enhancer regions)

Heterozygous SHOX gene variation involving only the enhancer regions	*N*	Pathogenicity
Deletion of 16 contiguous probes (05642-L05096 to 10251-L24246) located the 3′ regulatory region downstream of the gene, carrying all SHOX regulatory elements (CNE3, CNE4, CNE5, CNE7, CNE8, CNE9)	2	Pathogenic
Deletion of 14 contiguous probes (05642-L05096 to 14697-L24245) located the 3′ regulatory region downstream of the gene (CNE3, CNE4, CNE5, CNE7, CNE8, CNE9)	1	Pathogenic
Deletion of 8 contiguous probes (05642-L05096 to 13297-L24253) located in the 3′ regulatory region of the gene (CNE3, CNE4, CNE5, CNE7, CNE8)	2	Pathogenic
Deletion of 3 contiguous probes (probe 18893_L25091 to 05646_L24249) located in the 3′ regulatory region of the gene (CNE7, CNE8)	26	Pathogenic
Deletion of 2 contiguous probes (18889-L25087 to 18885-L24430) located in the 5′ regulatory region of the gene (CNE-5, CNE-3)	2	Pathogenic
Deletion of 2 contiguous probes (05645_L05099 and 05646_L15507) located in the 3′ regulatory region of the gene (CNE9)	3	Pathogenic
Duplication of 12 contiguous probes (18893_L25091 to 10251_L24246) located in the 3’ regulatory region of the gene (CNE7, CN8, CNE9)	1	Pathogenic
Duplication of 4 contiguous probes (09333_L10292) in the PAR1 region of regulatory elements (CNE5, CNE3 CNE2) in 5′	2	Pathogenic
Duplication of a single probe (05642_L05096) located between CNE2 and CNE3 in 3′ of the gene	1	Likely pathogenic
Duplication of a single probe 18889_L25087 of the regulatory element CNE-5 in 5′ of the gene	1	Likely pathogenic
Duplication of CNE-3 in the 3′ of the gene	1	Likely pathogenic

Pathogenicity was confirmed if the variation involves the entire SHOX gene, has been previously linked to the Leri–Weill phenotype, or causes skeletal effects in experimental models (reviewed by Marchini et al) [7, 21].

### Statistical analysis

Descriptive statistics were performed: continuous variables were expressed as medians (5th and 95th percentiles), and qualitative variables as percentages. Comparisons between groups were performed using the Student's *t* test and the Mann–Whitney test for continuous variables, and the Chi-square test was used for discrete variables.

Receiver operating characteristic (ROC) curves were calculated to assess the trade-off between sensitivity and specificity of the radiological criteria, using SHOX-positive subjects (identified through MLPA or sequencing) and SHOX-negative subjects (showing no SHOX variation by MLPA and sequencing). The resulting cutoffs were chosen to ensure a sensitivity of at least 80% (to avoid missing many SHOX-positive cases) and a specificity of no less than 40% (to prevent an excessively high rate of unnecessary molecular screening).

To assess whether the identified cutoffs were robust, analyses were also performed by comparing:

- SHOX-positive subjects with gene variation within the coding region with SHOX-negative subjects by MLPA and sequencing, and SHOX-positive subjects with gene variation within the enhancer region with SHOX-negative subjects identified by MLPA and sequencing: this allowed us to test the robustness of the cutoffs for sensitivity for the 2 types of SHOX gene variation.
- SHOX-positive and -negative subjects identified by MLPA alone: this enabled us to evaluate the robustness of the cutoffs when only deletions and duplications could be detected, a strategy used in several studies [5, 14, 18].

Finally, multiple logistic regression analyses were conducted to identify the optimal set of radiological predictors for SHOX gene variation (by MLPA and sequencing). Variance inflation factor was used for diagnosing collinearity between variables. The Hosmer–Lemeshow statistics assessed the regression model's goodness of fit. *P* values <.05 were considered to be statistically significant.

We used SPSS Statistics v25 (IBM Corp., Armonk, NY), Jamovi software version 2.3.18.0 (The Jamovi Project, 2022; https://www.jamovi.org), and GraphPad Prism 9 (GraphPad Software Inc.).

## Results

### Characteristics of the subjects

Figure 1 shows the diagnostic process flowchart. Of the 266 subjects with apparently ISS, SHOX gene variation was identified by MLPA in 26 (9.8%) of them. Of the 63 children who were tested further by sequencing after being found to have no SHOX gene variation by MLPA, 4 were tested positive (6% of the MLPA-negative ISS subjects tested). Overall, the MLPA followed by sequencing (when the initial MLPA test was negative) process in children with ISS yielded a 15% positivity rate for SHOX gene variation (two-thirds identified by MLPA, a third by sequencing).

Tables 1 and 2 provide the list of the identified SHOX gene variants and their pathogenicity. A third of the variations involved the coding regions, while two-thirds occurred only in the enhancer regions. For the analyses of sensitivity/specificity, only children with a pathogenic SHOX gene variation (94% of SHOX-positive subjects) were included, while those with a likely pathogenic variation were excluded.

Table 3 presents the clinical and radiological characteristics of the 59 children with a pathogenic SHOX gene variation (SHOX-positive by MLPA or sequencing), the 59 children with no SHOX gene variation (SHOX-negative by MLPA and sequencing), and the 177 SHOX-negative subjects by MLPA only. Many clinical characteristics were similar between SHOX-positive and SHOX-negative subjects, including birth length, target height, BMI, height, and sitting height.

**Table 3:** Clinical and radiological characteristics of the subjects based on SHOX gene status

	Pathogenic SHOX variation by MLPA or sequencing	No SHOX variation by MLPA and sequencing	No SHOX variation by MLPA alone
Number of subjects	59	59	177
Males/females	33/26	32/27	97/80
Age (years)	10.2 (3 to 14)	9.8 (2.97 to 14)	10.9 (4.2 to 14.9)
Birth length (cm)	48 (45 to 52)	47 (42 to 52)	48.0 (44.2 to 51)
Mother's height (SDS)	−1.2 (−3.0 to 0.1)	−1.0 (−2.9 to 0.7)	−0.7 (−2.5 to 0.8)*^a^*
Father's height (SDS)	−0.9 (−2.3 to 1.1)	−0.9 (−2.2 to 0.5)	−0.8 (−1.8 to 1.0)
Target height (SDS)	−1.0 (−2.2 to 0.3)	− 1 (−2.3 to 0.2)	−0.7 (−2.1 to 0.50)
BMI (SDS)	−0.4 (−2.6 to 1.3)	−0.2 (−1.8 to 1.4)	−0.5 (−2.9 to 1.2)
Height (SDS)	−2.0 (−3.0 to −0.4)	−2.2 (−3.5 to −1.0)	−2.2 (−3.2 to −1.2)
Sitting height/height	0.541 (0.501 to 0.582)	0.531 (0.487 to 0.570)	0.527 (0.494 to 0.574)
Sitting height/height >0.555	31%	15%*^a^*	13%*^a^*
Arm spam/height	0.979 (0.929 to 1.02)	0.968 (0.930 to 1.03)	0.991 (0.941 to 1.03)*^a^*
Arm spam/height <0.965	29%	44%	18%
Leg length + arm span/sitting height (binder ratio)*^c^*	2.63 (2.26 to 2.97)	2.74 (2.43 to 3.11)	2.77 (2.44 to 3.10)
Percentage of children with binder ratio < −1 SD score*^d^*	27%	18%	11%*^a^*
Parental short stature*^e^*	42%	26%*^a^*	18%*^b^*
**Radiological characteristics**			
Triangularization index	0.13 (0.06 to 0.24)	0.17 (0.06 to 0.29)	0.16 (0.06 to 0.27)
Carpal row angle (degrees)	120 (107 to 133)	129 (116 to 140)*^c^*	131 (115 to 145)*^c^*
Convexity of distal radial metaphysis (degrees)	135 (115 to 150)	147 (135 to 162)*^c^*	148 (132 to 165)*^c^*
Lucency of the ulnar border of the distal radius	13%	4%*^c^*	2.0%*^c^*

Median values (5th and 95th percentiles). *^a^P* < 0.05 vs SHOX positive by MLPA or sequencing; *^b^P* < 0.001 vs SHOX positive by MLPA or sequencing. *^c^*Binder ratio (see Refs. [5, 18]). *^d^*% of subjects with height > 110 cm and Binder Ratio < −1 SD score, according to Binder [5, 18]. *^e^*Height < 152 cm for women and < 163 cm for men.

The proportion of subjects with a sitting height-to-height ratio greater than 0.555 (the cutoff value proposed by Rappold et al) was significantly higher in the SHOX-positive group. The ROC curve AUC of the sitting height-to-height ratio was 0.66 (95% CI 0.54 to 0.78; *P* < .05). The sensitivity/specificity of the 0.555 cutoff value was 34% (95% CI 20%-52%) and 86% (95% CI 74%-93%), respectively.

In contrast, the proportion of subjects with an arm span-to-height ratio below 0.965, as proposed by Rappold et al, remained comparable between groups, and the ROC curve AUC of the arm span-to-height ratio was not significant, indicating no discriminating power of this ratio.

We calculated the (leg length + arm span)/sitting height ratio, as proposed by Binder et al [5], for children taller than 110 cm (79% of the cohort). We found that 18% of SHOX-negative subjects had a ratio < −1DS, consistent with the expected result under the hypothesis of a normal distribution. Twenty-seven percent of SHOX-positive subjects had a ratio < −1DS (see Table 3). Among those, 40% of patients with a SHOX gene variation in the coding region had a ratio < −1DS, compared with 21% of patients with a mutation in enhancer regions (see Table 4).

**Table 4:** Clinical and radiological characteristics of the subjects positive for a pathogenic SHOX gene variation according to the localization of the variation

	Enhancer region	Coding region	*P* value
Number of subjects (%)	39 (66)	20 (34)	
Age (years)	9.8 (4.2 to 14.2)	10.7 (1.6 to 12.1)	.250
Birth length (cm)	48 (45 to 52)	48 (46 to 50)	.653
Mother's height (SDS)	−1.0 (−2.4 to 0.1)	−1.6 (−3 to 0)	.042
Father's height (SDS)	−0.6 (−2 to 1.1)	−1.2 (−2.3 to 0.4)	.061
Target height (SDS)	−0.8 (−2.2 to 0.3)	−1.3 (−2.3 to 0.2)	.016
BMI (SDS)	−0.4 (−2.2 to 1.2)	0.3 (−3.1 to 1.3)	.997
Height (SDS)	−2.1 (−3.0 to −1.0)	−2.0 (−3.0 to −0,8)	.681
Sitting height/height	0.540 (0.504 to 0.583)	0.544 (0.503 to 0.575)	.652
Sitting height/height >0.555	23%	50%	.116
Arm spam/height	0.976 (0.930 to 1.02)	0.967 (0.916 to 1.01)	.521
Arm spam/height <0.965	21%	56%	.044
Leg length + arm span/sitting height (Binder ratio)*^a^*	2.67 (2.22 to 2.99)	2.65 (2.32 to 2.84)	.42
Percentage of children with Binder ratio < −1 SD score*^b^*	21%	40%	.17
Parental short stature*^c^*	37%	56%	.18
Radiological characteristics			
Triangularization index	0.13 (0.06 to 0.24)	0.15 (0.09 to 0.23)	.868
Carpal row angle (degrees)	120 (108 to 136)	119 (108 to 128)	.722
Convexity of distal radial metaphysis (degrees)	136 (116 to 151)	130 (115 to 146)	.045
Lucency of the ulnar border of the distal radius	21%	35%	.247

Median values (5th and 95th percentiles). *^a^*Binder ratio (see refs. [5, 18]) *^b^*Percentage of subjects with height > 110 cm and binder ratio < −1 SD score, according to binder [5, 18]. *^c^*Height < 152 cm for women and < 163 cm for men.

Regarding the radiological characteristics, the pyramidalisation of the carpal row, the median convexity of the distal radial metaphysis, and the percentage of subjects showing lucency at the ulnar border of the distal radius were significantly different between the SHOX-positive and SHOX-negative groups. However, no significant difference was observed in the triangularization index of the distal radial epiphysis. No clinical or radiological differences were found between boys and girls with SHOX gene variation. Notably, the triangularization index was negatively associated with age in both SHOX-positive (*P* < .001) and SHOX-negative (*P* < .001) subjects, while pyramidalization of the carpal row and the convexity of the distal radial metaphysis showed no relation to age (*P* = NS).

Table 4 compares patients with variations in the enhancer region to those with variations in the coding region. We found a higher proportion of subjects with an arm span-to-height ratio <0.965 among those with SHOX gene variation in the coding region, and a significantly more severe convexity angle of the distal radial metaphysis; however, no differences were observed for the other radiological criteria.

Convexity of the distal radial metaphysis could be measured in all patients, while the pyramidalization of the carpal row could not be assessed in most patients under 7 years old due to the lack of ossification of the scaphoid. Additionally, the triangularization of the distal radial epiphysis could not be measured in patients under 4 years old.

### ROC curves for the bone indices

**Figure 3:**
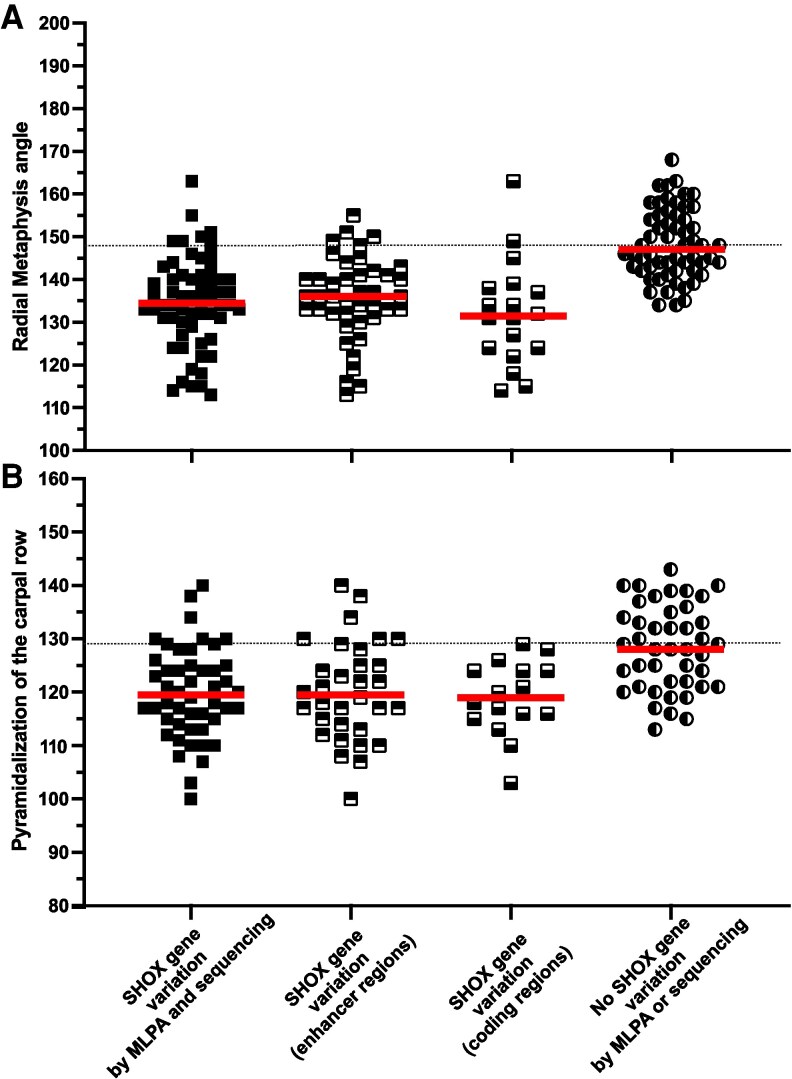
Scatter plots of the angle of the distal radial metaphysis convexity (A) and the pyramidalization of the carpal row (B) for the subjects with SHOX gene variation by MLPA or sequencing (true positive), SHOX gene variation within the enhancer region, and SHOX gene variation within the coding region, and no SHOX gene variation by MLPA and sequencing (true negative). Dashed line in (A) showed the cutoff angle of 147° for the distal radial metaphysis convexity, and 128° for the pyramidalization of the carpal row in (B).

The ROC curve AUC for the bone indices and the sensitivity/specificity of the cutoff points are reported in Table 5 (Fig. 3).

**Table 5:** ROC curves for the convexity of the distal radial metaphysis, the pyramidalization of the carpal row, and the triangularization of the distal radial epiphysis for differentiating ISS subjects with and without SHOX gene variation

Period	ROC AUC (95% CI)	Cutoff	Sensitivity (%) (95% CI)	Specificity (%) (95% CI)
**SHOX gene variation by MLPA and sequencing (+/−)**				
Convexity of the distal radial metaphysis	0.86*^b^* (95% CI 0.79-0.93)	≤147°	89% (95% CI 79%-95%)	50% (95% CI 38%-62%)
Pyramidalization of the carpal row	0.77*^b^* (95% CI 0.68-0.87)	≤128°	86% (95% CI 73%-93%)	49% (95% CI 35%-63%)
Triangularization of the distal radial epiphysis	0.62*^a^* (95% CI 0.51-0.73)	≤0.17	64% (95% CI 51%-76%)	53% (95% CI 39%-66%)
**SHOX gene variation in the coding regions (+/−)**				
Convexity of the distal radial metaphysis	0.90*^b^* (95% CI 0.78-1.0)	≤147°	94% (95% CI 73%-100%)	50% (95% CI 38%-62%)
Pyramidalization of the carpal row	0.80*^b^* (95% CI 0.68-0.91)	≤128°	93% (95% CI 70%-100%)	49% (95% CI 35%-63%)
Triangularization of the distal radial epiphysis	NS			
**SHOX gene variation in the enhancer regions (+/−)**				
Convexity of the distal radial metaphysis	0.84*^b^* (95% CI 0.76-0.92)	≤147°	87% (95% CI 73%-94%)	50% (95% CI 38%-62%)
Pyramidalization of the carpal row	0.76*^b^* (95% CI 0.65-0.88)	≤128°	82% (95% CI 64%-92%)	49% (95% CI 35%-63%)
Triangularization of the distal radial epiphysis	NS			
**SHOX gene variation by MLPA (+/−)**				
Convexity of the distal radial metaphysis	0.85*^b^* (95% CI 0.80-0.91)	≤147°	91% (95% CI 80%-96%)	52% (95% CI 46%-58%)
Pyramidalization of the carpal row	0.77*^b^* (95% CI 0.69-0.85)	≤128°	82% (95% CI 64%-92%)	52% (95% CI 45%-59%)
Triangularization of the distal radial epiphysis	0.61*^a^* (95% CI 0.52-0.70)	≤0.17	66% (95% CI 52%-77%)	47% (95% CI 41%-54%)

^*a*^ *P* < .05; *^b^P* < .001.

Analyses were first performed with the 59 SHOX-positive subjects by MLPA or sequencing and the 59 SHOX-negative subjects by MLPA and sequencing (i.e, true negative subjects).

For the convexity of the distal radial metaphysis, a cutoff point of 147 degrees or less had a sensitivity of 89% and a specificity of 50%. For the pyramidalization of the carpal row, a cutoff point of 128 degrees (or less) had a sensitivity of 86% and a specificity of 49%. For the triangularization of the distal radial epiphysis, a cutoff point of 0.17 (or less) had a sensitivity of 64%, and a specificity of 53%.

We also assessed whether these cutoffs are valid for subjects with SHOX variants in the coding regions and those with variants only in the enhancer regions (Table 5). Sensitivities were higher (>90%) for variants in the coding regions than in enhancer regions (82-87%), with a similar specificity of about 50%.

We then determined whether these cutoffs are still valid when only MLPA is performed (comparing MLPA-positive and MLPA-negative subjects), as many studies detected only deletions in the SHOX gene region. The cutoffs were still robust, as sensitivities/specificities were very close to those determined above (Table 5, Fig. 3).

### Evaluation of Rappold score and Binder algorithm

The ROC curve AUC of the score described by Rappold in our population was nonsignificant (AUC = 0.42, P NS). The proposed cutoff score of 4 showed Se 36% (95% CI 25%-48%) and Sp 51% (95% CI 38%-63%). The selection algorithm proposed by Binder, which aimed to minimize the number of missed SHOX-positive subjects, resulted in better sensitivity (81%, 95% CI 69%-90%) but very low specificity (10%, 95% CI 4%-21%).

### Multivariate logistic regression analyses

We evaluated whether the combination of the convexity of the distal radial metaphysis and the pyramidalization of the carpal row improved the discriminatory power for predicting SHOX gene variation in subjects who underwent MLPA and SHOX sequencing. For a cutoff of *P* > .40, this combination correctly classified 91% of SHOX-positive and 70% of SHOX-negative subjects. Variance inflation factors showed no collinearity between variables.

The probability of SHOX gene variation was estimated using the following equation:


$$P\;(\mathrm{SHOX}\;\mathrm{gene}\;\mathrm{variation})\;=\frac{1}{1\;+\;e\;-(34.3\;-0.14\;x\;\mathrm{radial}\;\mathrm{metaphysis}\;\mathrm{convexity}\;-\;0.12\;x\;\mathrm{pyramidalization}\;\mathrm{of}\;\mathrm{the}\;\mathrm{carpal}\;\mathrm{row})\;}$$

The Hosmer–Lemeshow goodness-of-fit test was nonsignificant, indicating that the model fit the data adequately. Each increase of the radial metaphysis convexity angle by 1° decreased the likelihood of SHOX gene variation by 13% (95% CI 7%-19%), and each increase of the pyramidalization of the carpal row angle by 1° decreased the likelihood of SHOX gene variation by 11% (95% CI 5%-17%).

Notably, no clinical criteria improved the discriminating ability of these 2 radiological criteria.

## Discussion

Our study found that 15% subjects with otherwise unexplained short stature exhibited SHOX gene variation by MLPA and sequencing, consistent with the literature, albeit at the higher end of the published prevalence [7]. We identified significant differences in the convexity of the distal radial metaphysis, the pyramidalization of the carpal row, and the percentage of patients with lucency of the ulnar border of the distal radius between subjects with and without SHOX gene variation. No difference was found regarding the triangularization of the distal radial epiphysis. The analyses enabled us to determine cutoffs for the convexity of the distal radial metaphysis and the pyramidalization of the carpal row, achieving a fair sensitivity of approximately 90% with a mild specificity of around 50%, which increased to 70% when combined. Although there was no cutoff with 100% sensitivity and specificity, the proposed cutoff could potentially decrease the need for molecular studies by at least 50%, with a sensitivity of 90%. This may be advantageous when resources allocated for such studies are limited.

The SHOX gene codes for a protein that acts as an antiproliferative factor and regulates the balance between proliferation and apoptosis in the hypertrophic chondrocytes of the growth plate in long bones [7, 21, 22]. SHOX gene variations have been identified in approximately 3% (range, 1.7%-22%) of subjects with ISS [6, 14-16], vs 15% of short individuals in this study (a third in the coding regions, and two-thirds in the enhancer regions). Not all studies have screened for variations within enhancer regions [18, 23], and tools for studying the SHOX region varied across studies, some detecting only large deletions [7, 8, 18], which may contribute to the discrepancies in the published percentages. Indeed, studies using MLPA and sequencing identified SHOX gene variations in 7% to 20% of ISS subjects, consistent with our findings [9, 15, 16]. Location within enhancer regions accounted for 26% to 60% of SHOX gene variations in children with apparently ISS [4, 7, 15, 24-26], compared with 66% in our study. Our comprehensive screening for variations in the SHOX gene, including both coding and enhancer regions, without any predefined criteria in subjects with ISS, likely led to a higher percentage of subjects with variations in the enhancer regions. This, in turn, raised the overall percentage of individuals with ISS who have SHOX gene variations to the high end of what is reported in the literature [5-7, 9, 14-18, 23]. When gene variations occurred in the coding regions, we found a higher percentage of subjects with a decreased arm span-to-height ratio, together with a more severe convexity of the distal radial metaphysis. This suggests that our cutoffs would be valid (and even more sensitive and specific) if the study were restricted to affected subjects with SHOX alterations in the coding regions, as appears to be the case for the (leg length + arm span)/sitting height ratio described by Binder [5], which shows a higher percentage of subjects with a ratio < −1DS in patients with a SHOX gene variation in the coding region (40% vs 21%, see Table 4). Accordingly, Rosilio et al found less severe phenotypes in patients with variations within the enhancer regions [15], while Benito-Sanz et al found that variations in the enhancer regions seem to cause phenotypes similar to those within the coding region [26].

Numerous studies indicate that SHOX gene variation is associated with variable phenotypes [3-5, 8]. We found no difference in height, target height, or BMI between children with and without SHOX gene variation; however, the percentage of patients with SHOX gene variation who had shorter limbs was higher. The difference in the sitting height-to-height ratio was not significant enough to discriminate between affected and unaffected children. In our population, the evaluation of the scores described by Rappold found sensitivities for a score greater than 4 or greater than 7, the 2 proposed cutoffs in the original study, to be 35% and 13%, respectively, compared with 71% and 61% in the cohort by Rappold. This can likely be explained by the fact that our population consisted of children with less severe phenotypes than those in the study by Rappold and that we also screened for SHOX gene variation within the enhancer regions in addition to coding regions [6]. Notably, body proportions depend on age, and while fixed cutoffs are easier to use, they may lack sensitivity and specificity because they are independent of age [27]. Similarly, the decision algorithm by Binder et al achieved high sensitivity (94%) but very low specificity (10%), supporting a systematic screening of SHOX gene variation.

Our study presents several limitations. First, sequencing was not performed in all patients with a negative MLPA result, and we may have missed point mutations. However, our proposed cutoffs for radiological criteria, calculated from the true-positive and true-negative (by MLPA and sequencing) short subjects, were robust enough, even when applied to subjects studied only by MLPA. Second, while deletions in the coding regions and large deletions in the enhancer regions are usually considered pathogenic, some duplications in the enhancer regions may not be [7, 25]. Although this topic remains under discussion due to the lack of functional studies, in clinical practice, detecting a SHOX variation in a short child may suggest the potential for rhGH treatment. Third, we were unable to find cutoffs for radiological criteria with 100% sensitivity while maintaining a fair specificity. Last, age was a limitation for radiological measurements: pyramidalization of the carpal row could not be measured in patients under 7 years, and only the convexity of the distal radial metaphysis could be used in younger patients. This underlined the importance of repeating X-rays in patients explored for ISS before the age of 7. Although inter- and intra-observer agreements for angle measurements were good, as indicated by Cronbach's alpha values, it is likely that the process of measuring these angles could be even easier and more consistent with the aid of artificial intelligence in the near future [28].

In conclusion, we identified cutoffs for diagnosing SHOX gene variation based on simple measurements from the left hand and wrist radiology. These cutoffs for distal radial metaphysis convexity and carpal row pyramidalization demonstrated fair sensitivity (90%) and moderate specificity (50%), which increased to 70% when combined. This indicates that in settings where molecular testing is accessible, routine screening with MLPA and sequencing in children with otherwise unexplained short stature is essential for identifying all SHOX gene variations.

## Data Availability

Some or all datasets generated during and/or analyzed during the current study are not publicly available but are available from the corresponding author on reasonable request.
